# Using Rotational Thromboelastometry (ROTEM) to Evaluate Coagulation Status After Intrapleural Recombinant Tissue Plasminogen Activator (rtPA) Administration in Regional Anesthesia

**DOI:** 10.7759/cureus.75547

**Published:** 2024-12-11

**Authors:** Alexander DeLeon, Luminita Tureanu, Olivia Vetter, Tiffany Liu, Rechna Korula, Vicente Garcia Tomas, Yogen Asher

**Affiliations:** 1 Anesthesiology, Northwestern University Feinberg School of Medicine, Chicago, USA

**Keywords:** paravertebral blocks, postoperative pain, rotem, thoracic epidural analgesia, thrombolytics

## Abstract

We report a case of a 45-year-old male who underwent thoracotomy for empyema and received multiple doses of intrapleural recombinant tissue plasminogen activator (rtPA). Given the recent administration of rtPA, the acute pain service performed a rotational thromboelastometry (ROTEM) test to assess coagulation before proceeding with regional anesthesia. The ROTEM results indicated normal to hypercoagulable clotting parameters, with a normal extrinsic thromboelastometry (EXTEM) lysis index at 30 minutes, suggesting no systemic effects of rtPA. Based on these findings, a single-shot paravertebral block was successfully performed, and subsequent pain management included a thoracic epidural without complication or evidence of spinal hematoma. This case demonstrates that ROTEM can provide valuable reassurance on coagulation status in patients who have received intrapleural rtPA, helping to assess the safety of regional anesthesia.

## Introduction

Intrapleural recombinant tissue plasminogen activator (rtPA) is often used to treat parapneumonic effusions and empyema [1]. rtPA is systemically known to cause coagulopathy. Post-thoracic surgical pain is frequently treated with regional anesthetic techniques, including paravertebral blocks, erector spinae blocks, or thoracic epidurals (TEPI) [2]. The American Society of Regional Anesthesia (ASRA) has published guidelines for using neuraxial techniques and deep peripheral nerve blocks with systemic anticoagulation [3]. ASRA recommends against the performance of spinal or epidural anesthetics except in "highly unusual circumstances." The ASRA guidelines primarily address subcutaneous and intravenous use of anticoagulants and do not specifically mention uses such as intrapleural administration of medications.

The concern with neuraxial blockade and paravertebral blocks in the setting of coagulopathy is the risk of spinal hematoma or bleeding into the paravertebral space [3]. The ASRA guidelines suggest a 48-hour time interval after intravenous rtPA administration in conjunction with normalization of clotting studies. Rotational thromboelastometry (ROTEM) is a viscoelastic test performed on whole blood or plasma that provides information on fibrinolysis, which is unavailable with traditional blood tests such as prothrombin time, partial thromboplastin time, fibrinogen, and platelet counts [4]. ROTEM has not been widely reported to be used as a tool to ensure normal coagulation to reduce the risk of spinal hematoma in the setting of intrapleural rtPA.

We present a case of a patient who underwent thoracoscopic surgery, which was converted to an open thoracotomy for the treatment of empyema utilizing intrapleural rtPA. The patient subsequently required additional pain management on POD#1 and required consultation with the acute pain service. A ROTEM test was performed before offering a single-shot paravertebral block to confirm normal coagulation.

## Case presentation

**Figure 1:**
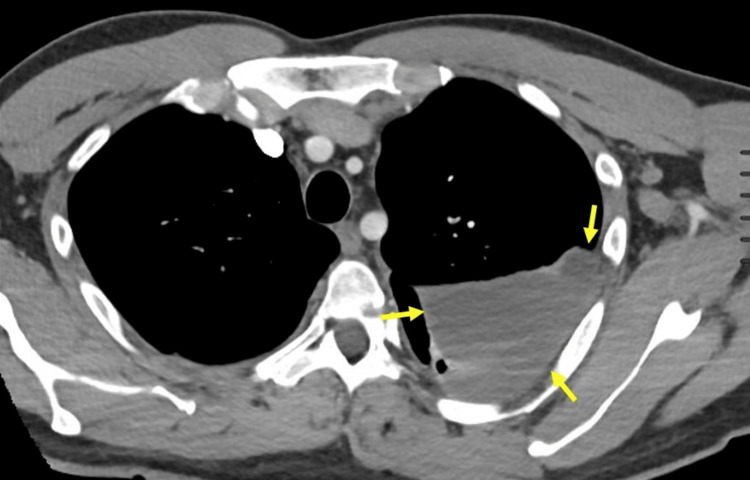
A non-contrast thoracic CT scan demonstrates a large left-sided loculated pleural effusion (yellow arrows) CT: computed tomography

We present a 45-year-old male with a history of hypertension who complained of shortness of breath for three weeks. The patient was found to have viral pneumonia with a loculated left parapneumonic effusion (Figure 1). On admission, the patient was also found to have elevated liver enzymes with an alanine transaminase of 70 u/L (reference range 0-52 u/L), aspartate aminotransferase 54 u/L (reference range 0-39 u/L), and alkaline phosphatase 158 u/L (reference range 34-104 u/L). On hospital day one (HD#1), the patient underwent the placement of a 14-Fr chest tube by the interventional pulmonology team, which subsequently drained 300 mL of serosanguinous fluid. Two hours later, 10 mg of rtPA (Activase, Genentech, San Francisco, California) was diluted in 30 mL of saline and administered through the chest tube. The patient received a second and third dose of intrapleural rtPA, 10 mg, over the next two days. He subsequently underwent thoracoscopy, which converted to an open thoracotomy on HD#5 for decortication, pleurectomy, chest tube placement, and washout.

**Figure 2:**
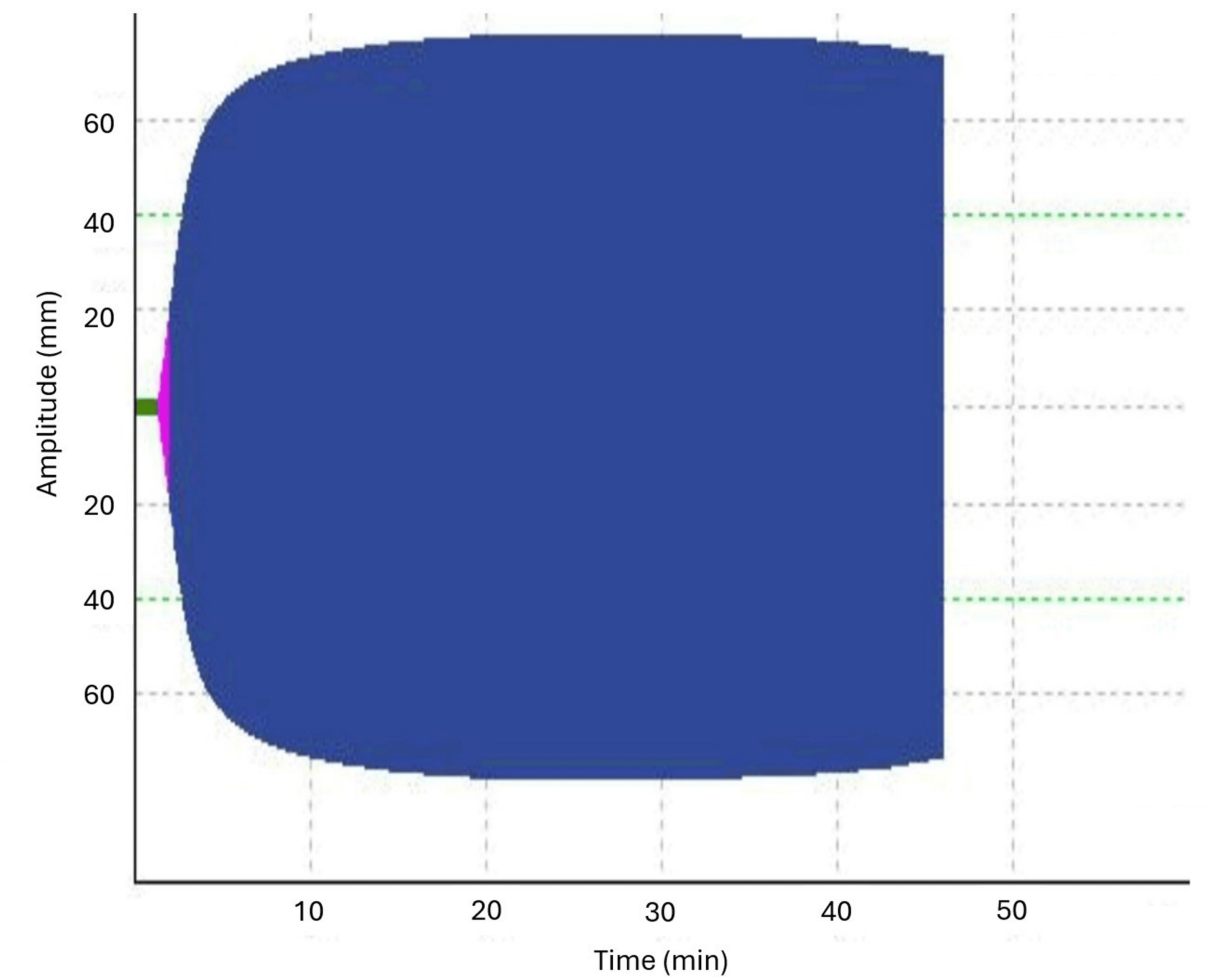
The EXTEM examination from the ROTEM demonstrates normal fibrinolysis over the 45-minute exam time EXTEM: extrinsic thromboelastometry, ROTEM: rotational thromboelastometry

On POD#1, the thoracic surgical team requested an anesthesia consultation for pain management. The patient complained of 10/10 pain, which was consistent with post-thoracic-surgical and chest tube pain, having failed conservative therapy. Given the history of receiving three doses of intrapleural rtPA as recently as two days prior, the acute pain team elected to obtain a ROTEM exam to assess coagulation status. Extrinsic thromboelastometry (EXTEM), a component of the ROTEM, results demonstrated a normal clotting time, a clot formation time of 39 seconds (low, reference range 48-127 seconds), an alpha angle of 82 degrees (high, reference range 65-80 degrees), and a slightly elevated amplitude 10 and 20 minutes after clotting time (Figure 2). The lysis index at 30 minutes after clotting time was 100%. The prothrombin time and international normalized ratio were within normal limits, and the platelet count was elevated to 590 (reference range 150 to 400 10^3/microL). The ROTEM indicated a normal to hypercoagulable profile, allowing for the safe placement of a deep nerve block or neuraxial analgesia.

The use of continuous pain catheters, including TEPI, was restricted to the medical floor location of the patient, so the acute pain team elected to perform single-shot paravertebral blocks under ultrasound guidance. A left-sided ultrasound-guided paravertebral block was placed at the T5 level using 20 mL of 0.25% bupivacaine (Sensorcaine-MPF, Fresenius Kabi USA, Melrose Park, IL). The patient's pain reduced from a 10/10 to a 4/10. The patient was transferred overnight to a post-surgical floor where continuous pain catheters could be managed.

On POD#2, a T7-8 TEPI was placed and dosed with 0.2% bupivacaine at 4 mL per hour. The patient's pain was well controlled, reporting pain scores of 2/10 post-procedure from 5/10, with a report of the patient being satisfied with his pain relief. There was no evidence of abnormal bleeding or spinal hematoma, and the patient recovered uneventfully.

## Discussion

Multiple modalities are available for acute pain management after thoracotomy, yet it is widely accepted that regional techniques are advantageous [2,5]. One of the contraindications to regional anesthesia is the concurrent use of systemic anticoagulation [3]. Intrapleural thrombolytic agents such as rtPA treat empyema and loculated pleural effusions [1]. The systemic effects of intrapleural rtPA have been reported as negligible, yet given the potentially devastating consequences of a spinal hematoma, additional caution is warranted [6].

The authors of the current case report were faced with the treatment dilemma of intractable post-thoracotomy pain in the setting of three separate doses of intrapleural rtPA. Non-interventional methods, such as multimodal pain medications, were unsuccessful. The ASRA guidelines for the safe utilization of regional techniques in the setting of anticoagulation mention using coagulation studies, clinical judgment, and risk-benefit when evaluating whether or not to proceed [3]. The published guidelines often do not explicitly state specific tests and cutoffs [3]. Given the unknown risk for epidural hematoma and the elective nature of interventional acute pain procedures, without ASRA guidance regarding intrapleural rtPA or laboratory reassurance, the acute pain team would have elected not to perform deep interventional pain therapies such as neuraxial analgesia. Thus, ROTEM provided a global and specific evaluation of the patient's clotting with the unique utility of evaluating the fibrinolytic status [4,7,8].

The authors of the current case report reasoned that a normal or hypercoagulable ROTEM would argue for the safety of a neuraxial technique, assuming the patient was amenable. The specific parameter of concern was the EXTEM lysis index at 30 minutes. Use of the lysis index at 30 minutes indicates whether the patient was experiencing the effects of systemic rtPA, a thrombolytic agent [4]. In the presented case, the lysis index at 30 minutes was normal (100%, reference range 95-100%), and all other indicators trended toward hypercoagulability, which is consistent with a postoperative patient with no systemic effects of rtPA.

When evaluating the impact of rtPA in a reduced lysis index, the aprotinin thromboelastometry (APTEM) lends information [4]. An APTEM is performed by adding aprotinin or tranexamic acid (TXA) to the sample to assess the effect of reversing thrombolysis. If the lysis index normalizes or improves with the aprotinin or TXA, abnormal fibrinolysis is suspected to be due to a thrombolytic mechanism [4,8]. Given the normal lysis 30 times with the EXTEM, the APTEM evaluation was less contributory, yet it was similarly indicative of a hypercoagulable state.

## Conclusions

Given the risk of a devastating spinal hematoma, the treatment of loculated empyemas with rtPA necessitates caution when considering postoperative neuraxial analgesia. Further research into the effects of intrapleural rtPA on systemic coagulation is required, which would help guide clinicians' decision-making processes regarding the safety of neuraxial or deep peripheral nerve blockade in patients who have received intrapleural rtPA. Without ASRA guidelines regarding intrapleural recombinant rtPA, ROTEM could provide further reassurance of normal coagulation before initiating regional anesthesia.
